# 5-(4-Meth­oxy­phen­yl)-3-phenyl­iso­thia­zole

**DOI:** 10.1107/S2414314626010151

**Published:** 2026-10-02

**Authors:** A. S. Jeevan Chakravarthy, V. Manushree, H. J. Yashwanth, N. R. Sreenatha

**Affiliations:** a Department of Chemistry BMS Institute of Technology & Management Autonomous under Visvesvaraya Technological University, Avalahalli Yelahanka Bengaluru 560064 Karnataka India; b Department of Microbiology, Krupanidhi Degree College, Bangalore, Karnataka 560035, India; c Department of Physics, Nitte Meenakshi Institute of Technology, Nitte (Deemed to be University), Bengaluru 560 064, Karnataka, India; d Research Center, Department of Physics, Government Engineering College, Bedarapura, Chamarajanagara 571313, Karnataka, India; University of Aberdeen, United Kingdom

**Keywords:** iso­thia­zole, dihedral angle, crystal structure

## Abstract

In the title compound, C_16_H_13_NOS, the dihedral angles between the central iso­thia­zole ring and pendant phenyl and *p*-meth­oxy­phenyl rings are 41.57 (15) and 4.63 (13)°, respectively. In the extended structure, weak C—H⋯π inter­actions may help to consolidate the packing.

## Abstract 2

In the title compound, the dihedral angles between the central iso­thia­zole ring and pendant phenyl and *p*-meth­oxy­phenyl rings are 41.57 (15) and 4.63 (13)°, respectively. In the extended structure, weak C—H⋯π inter­actions may help to consolidate the packing.

## Graphical abstract

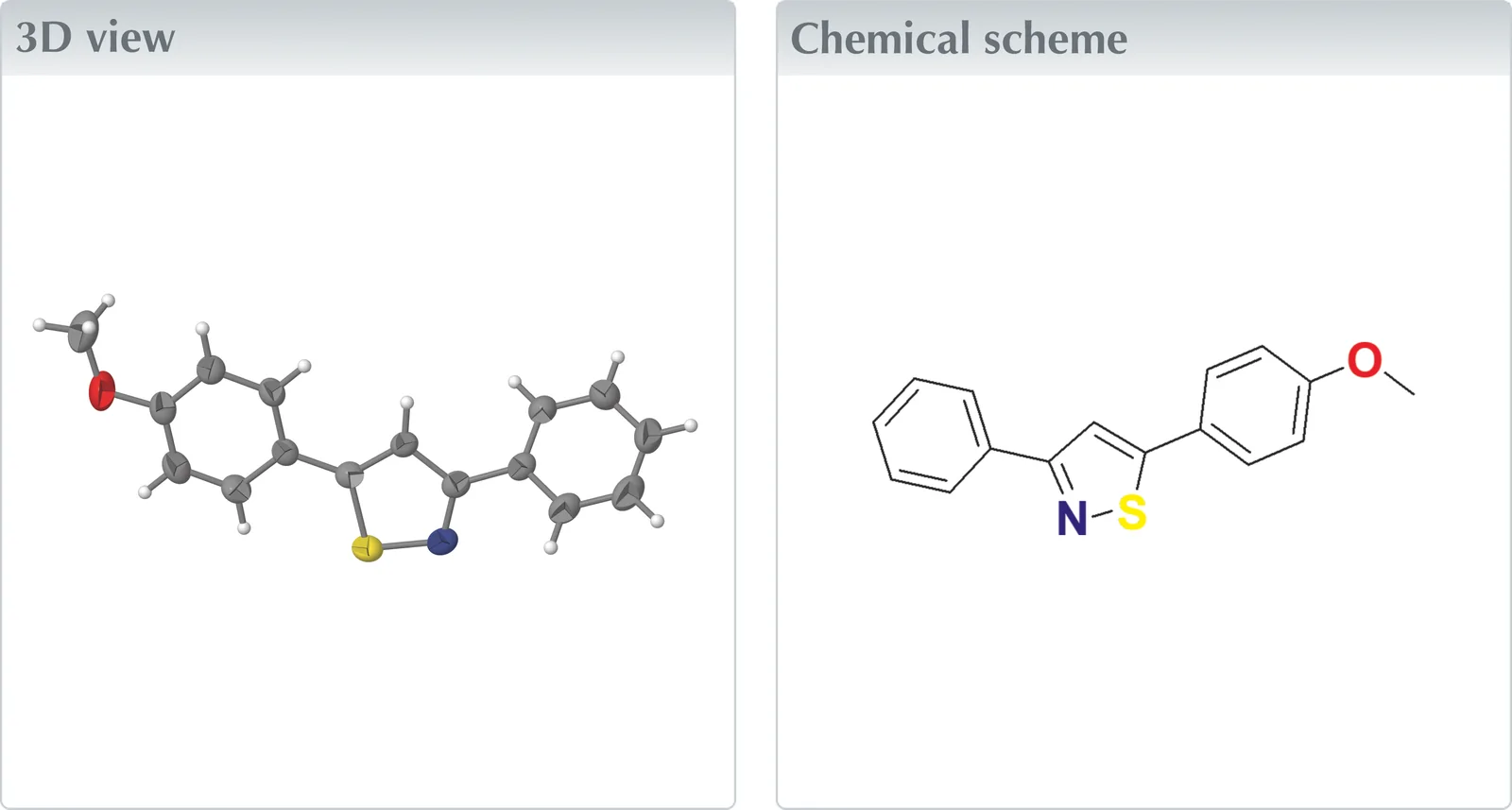

## Structure description

**Figure 1:**
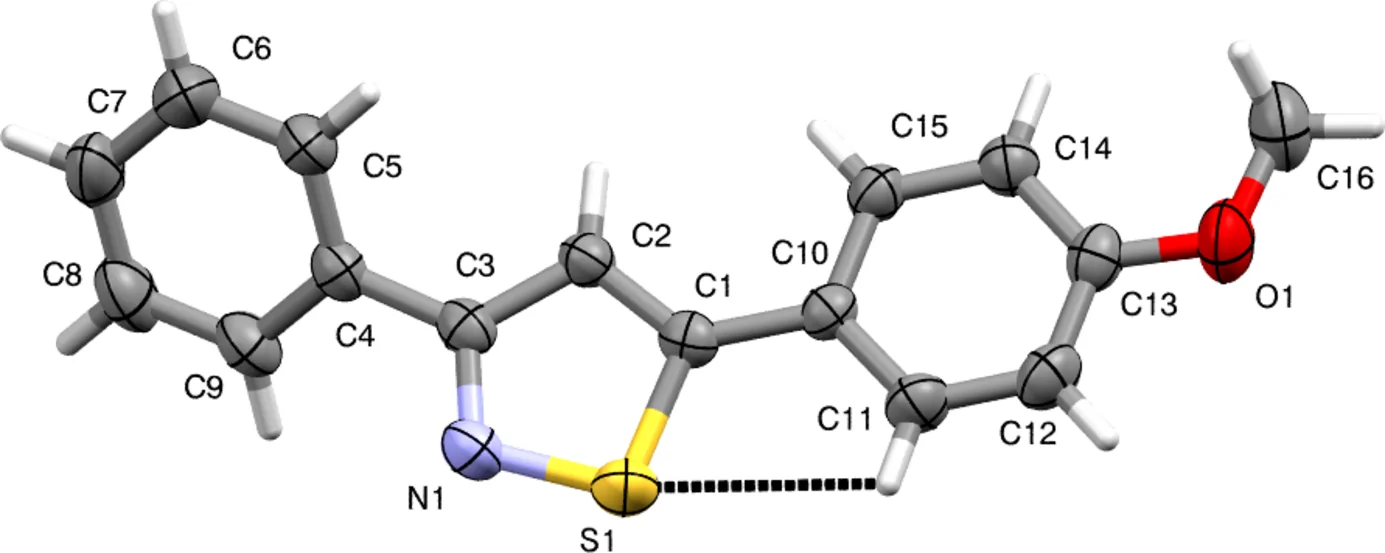
The mol­ecular structure of **2** with displacement ellipsoids drawn at the 50% probability level. The short C—H⋯S contact is shown as a dashed line.

The title compound, C_16_H_13_NOS (**2**), crystallizes with one mol­ecule in the asymmetric unit (Fig. 1 ▸) in space group *P*2_1_. The dihdral angles between the central C1–C3/N1/S1 iso­thia­zole ring and pendant C4–C9 phenyl and C10–C15 *p*-meth­oxy­benzene rings are 41.57 (15) and 4.63 (13)°, respectively. This suggests the possibility of enhanced conjugation between the C1 and C10 rings, but the C1—C10 and C3—C4 bond lengths of 1.471 (3) and 1.474 (4) Å, respectively, are almost identical. A short intra­molecular C11—H11⋯S1 contact (Table 1 ▸) occurs. Otherwise, the bond lengths and angles of **2** are within the ranges reported for related structures in the literature (Akkurt *et al.*, 2015 ▸; Sreenatha *et al.*, 2018 ▸, 2021 ▸; Sukhonosova *et al.*, 2016 ▸; Meundaeng *et al.*, 2019 ▸).

**Figure 2:**
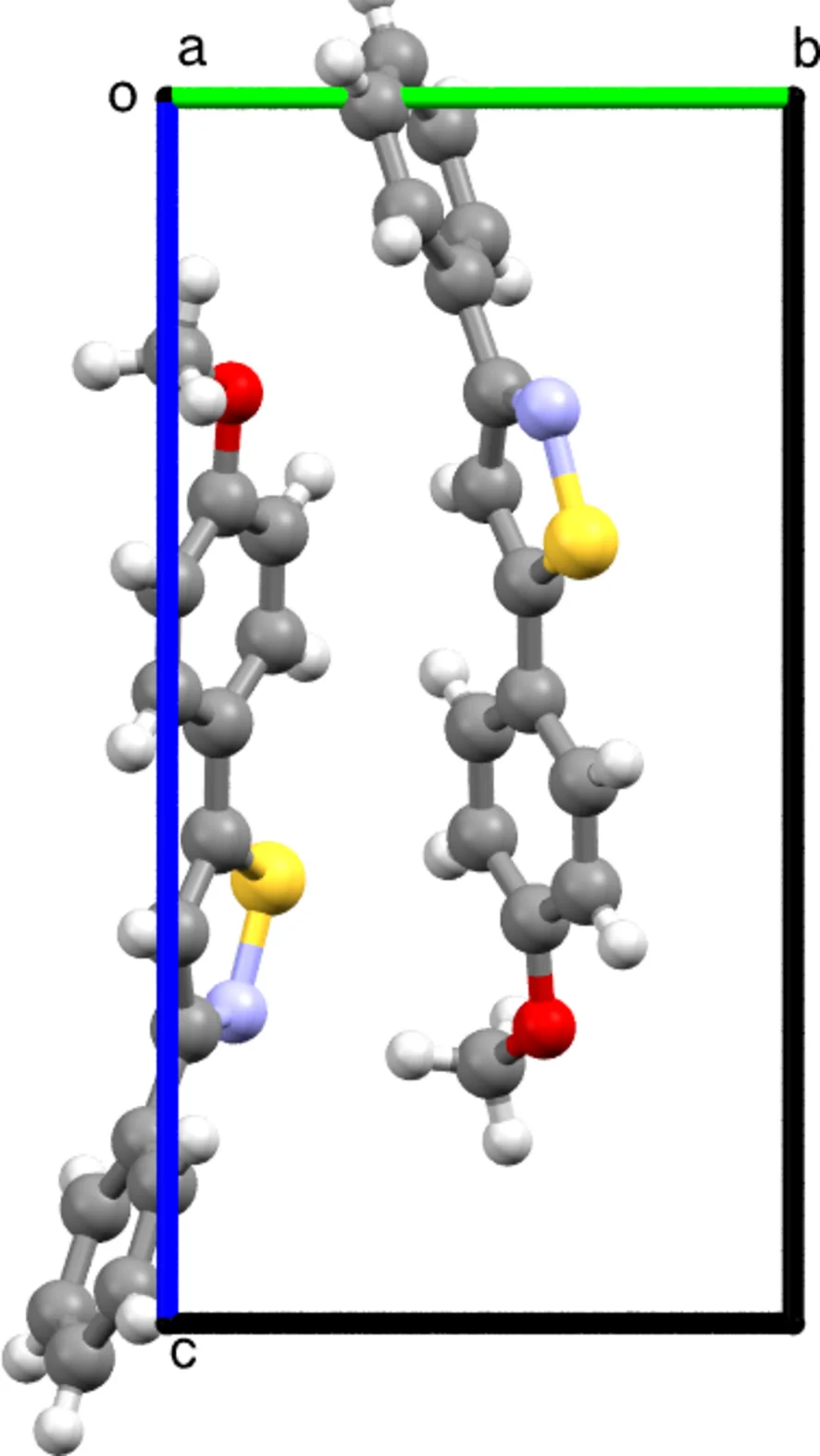
The packing of **2** viewed along the crystallographic *a* axis.

In the extended structure of **2**, weak C—H⋯π inter­actions (Table 1 ▸) may help to establish the packing (Fig. 2 ▸), with each ring accepting one such inter­action.

## Synthesis and crystallization

**Figure 3:**
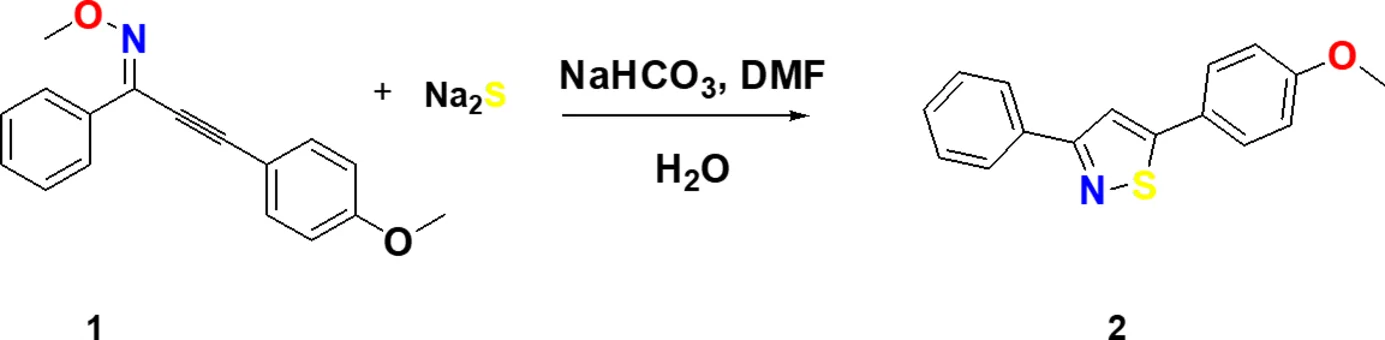
Synthesis scheme of the title compound.

The title compound was prepared by the literature method (Fig. 3 ▸) (Zhang *et al.* 2021 ▸) and 0.5 g of it was dissolved in 5 ml of *N*,*N*-dimethlformamide and was allowed to undergo slow evaporation to form colourless blocks of **2**.

## Refinement

Crystal data, data collection and structure refinement details are summarized in Table 2 ▸.

## Supplementary Material

Crystal structure: contains datablock(s) I. DOI: 10.1107/S2414314626010151/hb4573sup1.cif

Structure factors: contains datablock(s) I. DOI: 10.1107/S2414314626010151/hb4573Isup2.hkl

Supporting information file. DOI: 10.1107/S2414314626010151/hb4573Isup3.cml

CCDC reference: 2577952

Additional supporting information:  crystallographic information; 3D view; checkCIF report

## Figures and Tables

**Table 1:** Hydrogen-bond geometry (Å, °) *Cg*1, *Cg*2 and *Cg*3 are the centroids of the C1–C3/N1/S1, C4–C9 and C10–C15 rings, respectively.

*D*—H⋯*A*	*D*—H	H⋯*A*	*D*⋯*A*	*D*—H⋯*A*
C11—H11⋯S1	0.93	2.73	3.137 (3)	107
C8—H8⋯*Cg*2^i^	0.93	2.82	3.503 (4)	132
C12—H12⋯*Cg*1^ii^	0.93	2.88	3.542 (4)	129
C15—H15⋯*Cg*3^iii^	0.93	2.92	3.688 (3)	140

Symmetry codes: (i) 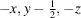; (ii) 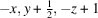; (iii) 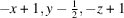.

**Table 2:** Experimental details

Crystal data	
Chemical formula	C_16_H_13_NOS
*M*_r_	267.33
Crystal system, space group	Monoclinic, *P*2_1_
Temperature (K)	298
*a*, *b*, *c* (Å)	6.0239 (2), 7.4924 (2), 14.7003 (5)
β (°)	92.779 (1)
*V* (Å^3^)	662.70 (4)
*Z*	2
Radiation type	Mo *K*α
μ (mm^−1^)	0.23
Crystal size (mm)	0.31 × 0.28 × 0.23

Data collection	
Diffractometer	Bruker SMART APEX
No. of measured, independent and observed [*I* > 2σ(*I*)] reflections	40942, 4059, 3351
*R*_int_	0.103
(sin θ/λ)_max_ (Å^−1^)	0.715

Refinement	
*R*[*F*^2^ > 2σ(*F*^2^)], *wR*(*F*^2^), *S*	0.047, 0.130, 1.01
No. of reflections	4059
No. of parameters	173
No. of restraints	1
H-atom treatment	H-atom parameters constrained
Δρ_max_, Δρ_min_ (e Å^−3^)	0.34, −0.23
Absolute structure	Flack *x* determined using 1295 quotients [(*I*^+^)−(*I*^−^)]/[(*I*^+^)+(*I*^−^)] (Parsons *et al.*, 2013 ▸)
Absolute structure parameter	0.13 (5)

Computer programs: *APEX* and *SAINT* (Bruker, 2012 ▸), *SHELXT2014/4* (Sheldrick, 2015*a* ▸), *SHELXL2019/2* (Sheldrick, 2015*b* ▸) and *PLATON*(Spek, 2009 ▸).

## full crystallographic data

### 5-(4-Methoxyphenyl)-3-phenylisothiazole Crystal data

**Table:**

C_16_H_13_NOS	*F*(000) = 280
*M**_r_* = 267.33	*D*_x_ = 1.340 Mg m^−^^3^
Monoclinic, *P*2_1_	Mo *K*α radiation, λ = 0.71073 Å
*a* = 6.0239 (2) Å	Cell parameters from 40942 reflections
*b* = 7.4924 (2) Å	θ = 3.1–30.5°
*c* = 14.7003 (5) Å	µ = 0.23 mm^−^^1^
β = 92.779 (1)°	*T* = 298 K
*V* = 662.70 (4) Å^3^	Block, colourless
*Z* = 2	0.31 × 0.28 × 0.23 mm

### 5-(4-Methoxyphenyl)-3-phenylisothiazole Data collection

**Table:**

Bruker SMART APEX diffractometer	*R*_int_ = 0.103
Radiation source: graphite	θ_max_ = 30.5°, θ_min_ = 3.1°
40942 measured reflections	*h* = −8→8
4059 independent reflections	*k* = −10→10
3351 reflections with *I* > 2σ(*I*)	*l* = −20→20

### 5-(4-Methoxyphenyl)-3-phenylisothiazole Refinement

**Table:**

Refinement on *F*^2^	Hydrogen site location: inferred from neighbouring sites
Least-squares matrix: full	H-atom parameters constrained
*R*[*F*^2^ > 2σ(*F*^2^)] = 0.047	*w* = 1/[σ^2^(*F*_o_^2^) + (0.0801*P*)^2^] where *P* = (*F*_o_^2^ + 2*F*_c_^2^)/3
*wR*(*F*^2^) = 0.130	(Δ/σ)_max_ = 0.001
*S* = 1.01	Δρ_max_ = 0.34 e Å^−^^3^
4059 reflections	Δρ_min_ = −0.23 e Å^−^^3^
173 parameters	Absolute structure: Flack *x* determined using 1295 quotients [(*I*^+^)-(*I*^-^)]/[(*I*^+^)+(*I*^-^)] (Parsons *et al.*, 2013)
1 restraint	Absolute structure parameter: 0.13 (5)

### 5-(4-Methoxyphenyl)-3-phenylisothiazole Special details

**Table:**

Geometry. All esds (except the esd in the dihedral angle between two l.s. planes) are estimated using the full covariance matrix. The cell esds are taken into account individually in the estimation of esds in distances, angles and torsion angles; correlations between esds in cell parameters are only used when they are defined by crystal symmetry. An approximate (isotropic) treatment of cell esds is used for estimating esds involving l.s. planes.
Refinement. Hydrogen atoms were placed in the calculated positions and refined as riding: C—H = 0.93Å with *U*_iso_(H) = 1.2*U*_eq_(C) for aromatic H atoms and C—H = 0.96 Å with *U*_iso_(H) = 1.5*U*_eq_(C) for methyl H atoms.

### 5-(4-Methoxyphenyl)-3-phenylisothiazole Fractional atomic coordinates and isotropic or equivalent isotropic displacement parameters (Å^2^)

**Table:**

	*x*	*y*	*z*	*U*_iso_*/*U*_eq_	
O1	0.4874 (4)	0.6019 (3)	0.75885 (14)	0.0623 (6)	
S1	−0.10260 (11)	0.66085 (10)	0.36254 (5)	0.0505 (2)	
N1	−0.0930 (4)	0.6076 (4)	0.25432 (17)	0.0473 (6)	
C1	0.1545 (4)	0.5777 (3)	0.39509 (17)	0.0355 (5)	
C2	0.2470 (4)	0.5106 (3)	0.31860 (17)	0.0369 (5)	
C3	0.1002 (4)	0.5291 (3)	0.24041 (17)	0.0375 (5)	
C4	0.1477 (4)	0.4669 (3)	0.14826 (17)	0.0391 (5)	
C5	0.3554 (5)	0.4910 (5)	0.11364 (19)	0.0482 (6)	
C6	0.3930 (6)	0.4388 (6)	0.0261 (2)	0.0627 (9)	
C7	0.2257 (6)	0.3597 (5)	−0.0282 (2)	0.0643 (10)	
C8	0.0206 (6)	0.3312 (5)	0.0068 (3)	0.0598 (8)	
C9	−0.0196 (5)	0.3850 (4)	0.0941 (2)	0.0490 (7)	
C10	0.2439 (4)	0.5804 (3)	0.49001 (17)	0.0351 (5)	
C11	0.1298 (5)	0.6657 (4)	0.55817 (19)	0.0461 (6)	
C12	0.2156 (5)	0.6693 (5)	0.64660 (19)	0.0503 (6)	
C13	0.4162 (5)	0.5883 (4)	0.66996 (18)	0.0430 (6)	
C14	0.5328 (5)	0.5032 (4)	0.60302 (18)	0.0427 (6)	
C15	0.4457 (5)	0.4996 (4)	0.51409 (18)	0.0403 (5)	
C16	0.6838 (8)	0.5155 (6)	0.7883 (2)	0.0706 (10)	
H2	0.387587	0.459462	0.318032	0.044*	
H5	0.469362	0.542442	0.149633	0.058*	
H6	0.532215	0.456678	0.002941	0.075*	
H7	0.251766	0.326248	−0.087616	0.077*	
H8	−0.091138	0.275419	−0.028633	0.072*	
H9	−0.158997	0.366739	0.117046	0.059*	
H11	−0.005761	0.720686	0.543607	0.055*	
H12	0.137634	0.726921	0.691119	0.060*	
H14	0.668691	0.449069	0.617911	0.051*	
H15	0.523906	0.441921	0.469656	0.048*	
H16A	0.665401	0.388862	0.781437	0.106*	
H16B	0.717252	0.543204	0.851271	0.106*	
H16C	0.803493	0.555074	0.752536	0.106*	

### 5-(4-Methoxyphenyl)-3-phenylisothiazole Atomic displacement parameters (Å^2^)

**Table:**

	*U*^11^	*U*^22^	*U*^33^	*U*^12^	*U*^13^	*U*^23^
O1	0.0806 (16)	0.0688 (15)	0.0369 (10)	0.0052 (13)	−0.0040 (10)	−0.0104 (10)
S1	0.0416 (3)	0.0599 (4)	0.0499 (4)	0.0159 (3)	0.0029 (3)	−0.0015 (3)
N1	0.0395 (12)	0.0548 (14)	0.0470 (13)	0.0073 (10)	−0.0044 (10)	0.0051 (10)
C1	0.0358 (11)	0.0307 (11)	0.0401 (12)	0.0005 (9)	0.0014 (9)	0.0026 (10)
C2	0.0332 (11)	0.0390 (12)	0.0382 (12)	0.0029 (9)	−0.0017 (9)	−0.0004 (10)
C3	0.0361 (12)	0.0353 (11)	0.0408 (13)	−0.0026 (9)	−0.0020 (10)	0.0030 (9)
C4	0.0394 (13)	0.0386 (12)	0.0387 (13)	0.0019 (10)	−0.0059 (10)	0.0020 (9)
C5	0.0391 (14)	0.0637 (17)	0.0414 (14)	0.0005 (12)	−0.0032 (11)	−0.0032 (13)
C6	0.0483 (18)	0.091 (3)	0.0487 (17)	0.0113 (17)	0.0045 (14)	−0.0061 (16)
C7	0.067 (2)	0.082 (2)	0.0428 (16)	0.0203 (18)	−0.0066 (14)	−0.0188 (16)
C8	0.0622 (19)	0.0598 (19)	0.0552 (17)	0.0033 (16)	−0.0200 (15)	−0.0146 (15)
C9	0.0443 (15)	0.0497 (15)	0.0518 (16)	−0.0033 (12)	−0.0106 (12)	−0.0038 (12)
C10	0.0373 (12)	0.0319 (10)	0.0364 (11)	−0.0007 (9)	0.0048 (9)	−0.0018 (9)
C11	0.0453 (13)	0.0452 (13)	0.0480 (14)	0.0094 (13)	0.0048 (10)	−0.0081 (12)
C12	0.0567 (16)	0.0499 (15)	0.0453 (14)	0.0038 (15)	0.0111 (12)	−0.0114 (13)
C13	0.0538 (15)	0.0388 (12)	0.0366 (12)	−0.0044 (11)	0.0049 (11)	−0.0038 (10)
C14	0.0434 (14)	0.0435 (13)	0.0412 (13)	0.0047 (11)	0.0009 (11)	−0.0016 (11)
C15	0.0426 (14)	0.0428 (13)	0.0361 (12)	0.0045 (11)	0.0062 (10)	−0.0037 (10)
C16	0.087 (3)	0.078 (2)	0.0453 (17)	0.003 (2)	−0.0114 (17)	0.0013 (17)

### 5-(4-Methoxyphenyl)-3-phenylisothiazole Geometric parameters (Å, º)

**Table:**

S1—N1	1.644 (3)	C15—H15	0.9300
S1—C1	1.716 (2)	C14—H14	0.9300
O1—C13	1.359 (3)	C12—H12	0.9300
O1—C16	1.399 (5)	C5—C4	1.385 (4)
N1—C3	1.329 (4)	C5—C6	1.374 (5)
C1—C2	1.374 (4)	C5—H5	0.9300
C1—C10	1.471 (3)	C4—C9	1.396 (4)
C3—C2	1.423 (3)	C9—C8	1.378 (5)
C3—C4	1.474 (4)	C9—H9	0.9300
C10—C15	1.389 (4)	C7—C8	1.378 (6)
C10—C11	1.397 (4)	C7—C6	1.387 (5)
C2—H2	0.9300	C7—H7	0.9300
C11—C12	1.376 (4)	C6—H6	0.9300
C11—H11	0.9300	C8—H8	0.9300
C13—C12	1.381 (4)	C16—H16A	0.9600
C13—C14	1.391 (4)	C16—H16B	0.9600
C15—C14	1.385 (4)	C16—H16C	0.9600
			
N1—S1—C1	96.22 (12)	C13—C12—C11	120.7 (3)
C13—O1—C16	119.1 (3)	C13—C12—H12	119.6
C3—N1—S1	109.17 (18)	C11—C12—H12	119.6
C2—C1—C10	129.6 (2)	C4—C5—C6	120.0 (3)
C2—C1—S1	107.52 (18)	C4—C5—H5	120.0
C10—C1—S1	122.92 (19)	C6—C5—H5	120.0
N1—C3—C2	115.7 (2)	C5—C4—C9	119.2 (3)
N1—C3—C4	119.5 (2)	C5—C4—C3	121.1 (2)
C2—C3—C4	124.8 (2)	C9—C4—C3	119.7 (3)
C15—C10—C11	118.2 (2)	C8—C9—C4	120.3 (3)
C15—C10—C1	120.7 (2)	C8—C9—H9	119.8
C11—C10—C1	121.1 (2)	C4—C9—H9	119.8
C1—C2—C3	111.4 (2)	C8—C7—C6	119.4 (3)
C1—C2—H2	124.3	C8—C7—H7	120.3
C3—C2—H2	124.3	C6—C7—H7	120.3
C12—C11—C10	120.7 (3)	C5—C6—C7	120.8 (3)
C12—C11—H11	119.6	C5—C6—H6	119.6
C10—C11—H11	119.6	C7—C6—H6	119.6
O1—C13—C12	115.9 (3)	C9—C8—C7	120.2 (3)
O1—C13—C14	124.6 (3)	C9—C8—H8	119.9
C12—C13—C14	119.4 (2)	C7—C8—H8	119.9
C10—C15—C14	121.3 (2)	O1—C16—H16A	109.5
C10—C15—H15	119.4	O1—C16—H16B	109.5
C14—C15—H15	119.4	H16A—C16—H16B	109.5
C13—C14—C15	119.7 (3)	O1—C16—H16C	109.5
C13—C14—H14	120.1	H16A—C16—H16C	109.5
C15—C14—H14	120.1	H16B—C16—H16C	109.5
			
C1—S1—N1—C3	−0.5 (2)	O1—C13—C14—C15	−179.1 (3)
N1—S1—C1—C2	0.9 (2)	C12—C13—C14—C15	−0.5 (4)
N1—S1—C1—C10	−177.9 (2)	C10—C15—C14—C13	0.4 (4)
S1—N1—C3—C2	−0.1 (3)	O1—C13—C12—C11	179.1 (3)
S1—N1—C3—C4	179.4 (2)	C14—C13—C12—C11	0.4 (5)
C2—C1—C10—C15	−3.4 (4)	C10—C11—C12—C13	−0.2 (5)
S1—C1—C10—C15	175.1 (2)	C6—C5—C4—C9	1.7 (5)
C2—C1—C10—C11	175.9 (3)	C6—C5—C4—C3	−177.2 (3)
S1—C1—C10—C11	−5.5 (4)	N1—C3—C4—C5	138.5 (3)
C10—C1—C2—C3	177.7 (2)	C2—C3—C4—C5	−42.1 (4)
S1—C1—C2—C3	−1.1 (3)	N1—C3—C4—C9	−40.4 (4)
N1—C3—C2—C1	0.8 (3)	C2—C3—C4—C9	139.0 (3)
C4—C3—C2—C1	−178.6 (2)	C5—C4—C9—C8	−0.9 (4)
C15—C10—C11—C12	0.0 (5)	C3—C4—C9—C8	178.0 (3)
C1—C10—C11—C12	−179.3 (3)	C4—C5—C6—C7	−0.8 (6)
C16—O1—C13—C12	176.8 (3)	C8—C7—C6—C5	−0.9 (6)
C16—O1—C13—C14	−4.6 (5)	C4—C9—C8—C7	−0.8 (5)
C11—C10—C15—C14	−0.2 (4)	C6—C7—C8—C9	1.7 (6)
C1—C10—C15—C14	179.2 (2)		

### 5-(4-Methoxyphenyl)-3-phenylisothiazole Hydrogen-bond geometry (Å, º)

*Cg*1, *Cg*2 and *Cg*3 are the centroids of the C1–C3/N1/S1, C4–C9 and C10–C15 rings, respectively.

**Table:**

*D*—H···*A*	*D*—H	H···*A*	*D*···*A*	*D*—H···*A*
C11—H11···S1	0.93	2.73	3.137 (3)	107
C8—H8···Cg2^i^	0.93	2.82	3.503 (4)	132
C12—H12···Cg1^ii^	0.93	2.88	3.542 (4)	129
C15—H15···Cg3^iii^	0.93	2.92	3.688 (3)	140

Symmetry codes: (i) −*x*, *y*−1/2, −*z*; (ii) −*x*, *y*+1/2, −*z*+1; (iii) −*x*+1, *y*−1/2, −*z*+1.

## References

[bb1] Akkurt, M., Smolenski, V. A., Mohamed, S. K., Jasinski, J. P., Hassan, A. A. & Albayati, M. R. (2015). *Acta Cryst.* E**71**, o776–o777. 10.1107/S2056989015017454 PMC4647441 26594474

[bb2] Bruker (2012). *APEX* and *SAINT*. Bruker AXS Inc., Madison, Wisconsin, USA.

[bb3] Meundaeng, N., Rujiwatra, A. & Prior, T. J. (2019). *Acta Cryst.* E**75**, 185–188. 10.1107/S2056989019000124 PMC6362641 30800448

[bb4] Parsons, S., Flack, H. D. & Wagner, T. (2013). *Acta Cryst.* B**69**, 249–259. 10.1107/S2052519213010014 PMC3661305 23719469

[bb5] Sheldrick, G. M. (2015*a*). *Acta Cryst.* A**71**, 3–8.

[bb6] Sheldrick, G. M. (2015*b*). *Acta Cryst.* C**71**, 3–8.

[bb7] Spek, A. L. (2009). *Acta Cryst.* D**65**, 148–155. 10.1107/S090744490804362X PMC2631630 19171970

[bb8] Sreenatha, N. R., Jeevan Chakravarthy, A. S., Lakshminarayana, B. N. & Hariprasad, S. (2021). *J. Mol. Struct.* **1225**, 129116, 1–8.

[bb9] Sreenatha, N. R., Lakshminarayana, B. N., Ganesha, D. P. & Gnanendra, C. R. (2018). *Acta Cryst.* E**74**, 1451–1454. 10.1107/S2056989018012173 PMC6176444 30319799

[bb10] Sukhonosova, E. V., Sokov, S. A., Ostapenko, G. I., Bunev, A. S., Dorovatovskii, P. V., Zubavichus, Y. V. & Khrustalev, V. N. (2016). *Acta Cryst.* E**72**, 1321–1325. 10.1107/S2056989016013104 PMC5120716 27920926

[bb11] Zhang, Z.-Z., Chen, R., Zhang, X.-H. & Zhang, X.-G. (2021). *J. Org. Chem.* **86**, 636–642.

